# Shower dehumidification to reduce nontuberculous mycobacteria aerosolization

**DOI:** 10.1186/s13104-024-06751-6

**Published:** 2024-03-28

**Authors:** Michael X. Kostecki, Yvonne L. Chan, Jennifer R. Honda

**Affiliations:** 1 ’Iolani School, 563 Kamoku St, 96826 Honolulu, Hawai’i USA; 2 Department of Cellular and Molecular Biology, School of Medicine, University of Texas Health Science Center at Tyler, 11937 US Hwy 271 BMR Building, 75708 Tyler, TX USA

**Keywords:** Nontuberculous mycobacteria, Shower, Aerosols, Dehumidification, Hawai’i

## Abstract

**Objective:**

Nontuberculous mycobacteria (NTM) are environmentally acquired opportunistic pathogens that can cause recalcitrant lung disease. Prior reports have demonstrated links between shower use and infections, yet the aerosolization of NTM from showerheads, as well as the humidity levels that may modulate NTM aerosolization from showerheads is less studied. The objective of the current study was to investigate the role of humidity in NTM aerosolization among showers in homes located in a geographic area with high lung disease incidence, Hawai’i, and test whether deployment of a dehumidifier in well-ventilated bathrooms reduce NTM exposure.

**Results:**

Across two sampling events and five showers, existing NTM showerhead biofilms along with shower air were sampled at three points: pre-shower, post-shower, and post-dehumidification. In each of the sampling events, respiratory relevant NTM species were identified from shower biofilms, which were also detected in aerosolized shower air after showering events, but not after the shower was dehumidified and bathrooms vented. While sample size was small, these data suggest running a shower is a possible source of NTM aerosolization and using a commercial household dehumidifier in conjunction with opening bathroom doors and windows may be simple, cost-effective interventions to reduce environmental NTM exposures.

**Supplementary Information:**

The online version contains supplementary material available at 10.1186/s13104-024-06751-6.

## Introduction

Nontuberculous mycobacteria (NTM), including species in the *Mycobacterium avium* complex (MAC), are environmental opportunistic pathogens found in freshwater systems [1]. NTM form biofilms on household piping and showerhead surfaces that are aerosolized and inhaled to cause chronic lung infections in susceptible individuals [2]. Other important NTM such as *Mycobacterium abscessus* are also effectively aerosolized and recovered in particles small enough to enter the human lung [3, 4].

In the United States (U.S.), Hawai’i shows the highest NTM lung disease prevalence rates [5]. Respiratory relevant NTM are common in Hawai’i household showers [6, 7] and environmental features such as high evapotranspiration rates and soil minerals have been reported to contribute to NTM abundance. Separately, the role of relative humidity (RH) to NTM aerosolization has been less studied. It’s been reported that aerosolized *M*. *avium* counts increase by 12-19% after incubation in a commercial humidifier compared to a 30 min exposure under non-humidified conditions [8]. Beyond this, evidence is scant, particularly in the context of the role of RH in NTM aerosolization from showerheads.

This study was performed to generate new data regarding the aerosolization of NTM from Hawai’i household showerheads. We also tested whether using a dehumidifier reduces NTM in showers as an environmental NTM intervention protocol. We hypothesized that removing water from the air (i.e., dehumidifying a shower) reduces NTM in homes in a U.S. geographic location where NTM lung disease is prevalent.

## Methods

### Sample size and home locations

A total of five showers from five O’ahu non-patient homes were included in this study (Additional Fig. 1). Each home was sampled across two sampling events. The first event was performed in December 2022 and environmental samples were cultured onto Middlebrook 7H10 OADC plates. The second event occurred 16 weeks later in April 2023 when the same set of environmental samples were collected but cultured onto Middlebrook 7H10 OADC plates supplemented with 1% malachite green. Permission was obtained from homeowners to perform environmental sampling. Homes did not have air conditioning and bathroom exhaust fans were turned off for the duration of the sampling period. Showers were not used within 12 h prior to sampling.

### Sampling strategy

To determine if showerheads were colonized by NTM, sterile, synthetic swabs were used to collect showerhead biofilms and microbiologically processed using cetylpyridinium chloride (CPC) decontamination methods as published [6, 7]. Middlebrook agar plates were inoculated with samples collected pre-CPC treatment and post-CPC treatment. Prior to air sampling, bathrooms were sealed by closing all windows and doors. Duplicate baseline samples of shower air were collected by placing a Surface Air System (SAS) Impaction Sampler (calibrated to collect 550 L of air over 3.1 minutes) 6’ directly below the showerhead (Additional Fig. 2A). Air samples were directly impacted onto 7H10 + OADC agar plates or 7H10 OADC plates + 1% malachite green to stringently inhibit mold overgrowth. In parallel, a hygrometer (ThermoPro TP50, i-Tronics, Reno, NV) was used to report the RH (% water vapor in the air). Then, the shower was turned on to monitor the water temperature and RH changes every minute for 8 min, the average shower time for Americans [9]. After 8 min of shower run, “post-shower” air samples were collected and microbiologically cultured.

To investigate the role of dehumidification on NTM aerosolization, a commercially available dehumidifier unit was placed in the shower and turned on (Additional Fig. 2B) with open bathroom windows and doors. Over 15–30 min, RH levels were measured until a 10% reduction in RH was achieved and the shower air was resampled onto 7H10 OADC agar plates or 7H10 OADC malachite green plates using the SAS Sampler. Plating onto agar plates was performed in duplicate; one plate was incubated at 30 °C and the other incubated at 37 °C for 17–21 days. Overall, a total of 50 culture plates were inoculated per sampling event (5 homes x 5 test samples x 2 incubation temperatures).

### NTM species and subspecies dentification by partial 16 S and rpoB gene sequencing

NTM-like colonies on agar plates were identified by first visually inspecting the colony’s morphology and by picking NTM-like colonies for direct DNA extraction. Colonies were added to silica-beads and TE solution, beat for 45 s, and heated for 10 min at 100 °C. DNA in the supernatant was collected for PCR. 16 S rRNA using primers 27 F and 1492R and *rpoB* gene sequencing were used for NTM identification. The 16 S rRNA forward primer sequence was AGAGTTTGATYMTGGCTCAG and reverse primer was TTACCGCCGCKGCTGGCAC. *rpoB* primers were CGACCACTTCGGCAACCG (forward) and TCGATCGGGCACATCCGG (reverse) [10]. *rpoB* primers were used at 0.6µM in a 25 µl reaction and 16 S primers were used at 0.3µM in a 15µL reaction. Both reactions used 2x Bioline BioMix Red Taq (Bio-25,006). PCR conditions: denaturation at 95 °C for 15 min, 30 cycles of DNA denaturation for 1 min, primer annealing at 61.5 °C for 1 min, extension at 72 °C for 1 min with a final extension step at 72 °C for 10 min [11]. PCR products were sent to Laragen Co. for Sanger Sequencing. Sequences were compared against type strain sequences deposited in the National Center for Biotechnology Information (NCBI) GenBank using the BLAST algorithm. Sequences can be found under NCBI GenBank accession numbers OR438667-OR438674 and OR428696-OR428698.

## Results

### RH increases with extended shower time

**Fig. 1:**
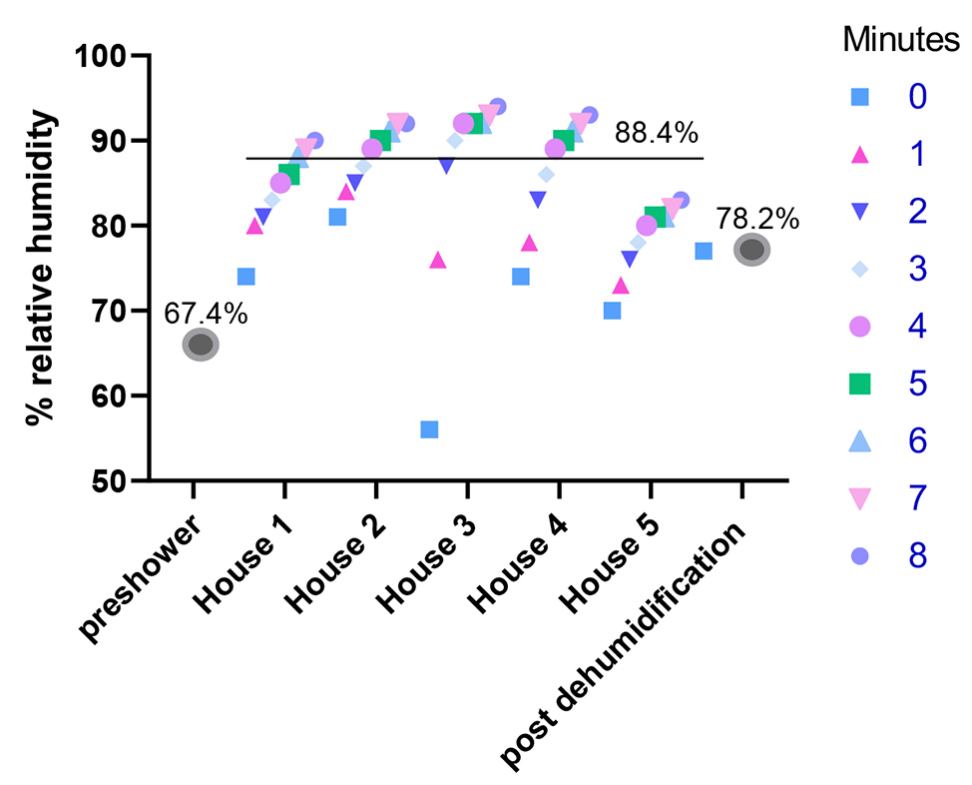
RH levels increase over an 8 min recording period after shower runs, but lowers after dehumidification. Ambient RH levels was measured using a hygrometer before the shower was turned on and averaged across the five homes sampled (“preshower”). Next, shower RH levels were recorded at each of the five homes at 1 min intervals for 8 min while the shower was running. A final RH measurement was recorded for each shower after dehumidification (“post dehumidification”), the average value is shown.

The average pre-shower RH was 67.4% ± SD 9.2% for the first sampling event (Fig. 1). Once the shower was turned on, the average RD increased to 88.4% ± SD 4.9% across the five houses sampled. After dehumidifying, the shower RH average lowered to 78.2% ± SD 4.8%. The average shower water temperature was 105 °F (∼ 40.5 °C). Similarly, the average humidities for pre-shower, post-shower, and post-dehumidification were 78.2% ± SD 4.7%, 90.6% ± SD 2.1%, 78.6% ± SD 3.3%, respectively for the second sampling event (Additional Fig. 3) with an average shower water temperature at 104 °F (40 °C).

### Shower dehumidification reduces aerosolized *Mycobacterium chelonae*

To understand whether NTM from showerheads were aerosolized during showering, we probed for colonizing NTM by first swab sampling the existing showerhead biofilm. Of the 50 culture plates, four were NTM positive (8%). From these four plates, nine NTM colonies were picked for identification. Five colonies (55%) were identified as *M. chelonae*, two (22%) *Mycobacterium porcinum*, and two (22%) *Mycobacterium intracellulare*. Overall, *M. chelonae* and *M. porcinum* were microbiologically identified from the duplicate plates that were incubated at 30 °C. These species were recovered from the CPC treated showerhead biofilm swab samples collected from House 2, but not any other House 2 plates. *M. intracellulare* was identified from the CPC treated showerhead biofilm swab sample collected from House 4, but not from any of the other samples from the same home.

We highlight the culture results of plates incubated at 37 °C in Table 1. *M. chelonae* was detected in one (House 1) out of the five (20%) showerheads sampled. *M. chelonae* was recovered from the air post shower in House 1, despite no detection of NTM in shower air before the shower was turned on for 8 min. Importantly, the air sample collected after House 1 shower stall was dehumidified was devoid of *M. chelonae* or other NTM. Of note, mold had grown on many of the SAS sampler plates.

**Table 1:** Dehumidification reduced *Mycobacterium chelonae* aerosolization, sampling round 1 (37 °C)

	Biofilm		Air		
House:	Showerhead biofilm (swab):	Showerhead biofilm (swab), post- disinfection	Pre shower air (SAS):	Post shower air (SAS):	Post dehumidification (SAS):
1	***M. chelonae***	No NTM	No NTM	***M. chelonae***	No NTM
2	No NTM	No NTM	No NTM*	No NTM*	No NTM*
3	No NTM	No NTM	No NTM	No NTM	No NTM
4	No NTM	No NTM	No NTM*	No NTM*	No NTM
5	No NTM	No NTM	No NTM*	No NTM*	No NTM

(*) Limitation - indicates instances where mold overgrowth likely reduced NTM detection

### Shower dehumidification reduces aerosolized *Mycobacterium intracellulare*

**Fig. 2:**
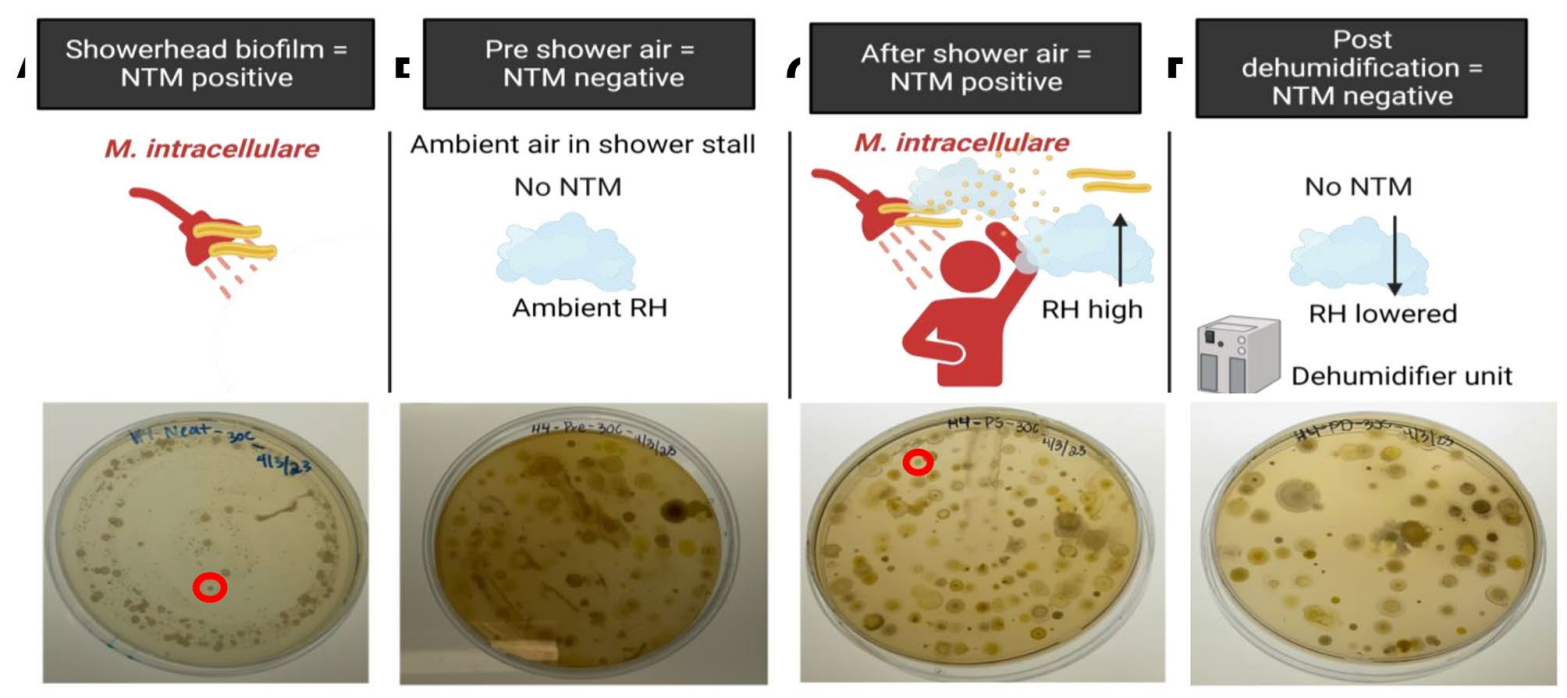
Shower aerosolized *M. intracellulare* is reduced post dehumidification in House 4, sampling event 2. **A)***M. intracellulare* (red circle) was detected in the agar plate inoculated with the showerhead biofilm sample of House 4. **B)** NTM was not detected in air samples collected in House 4 shower at ambient RH. **C)***M. intracellulare* (red circle) was recovered from the agar plate inoculated with air sample from House 4 after a showering event. **D)***M. intracellulare* and other NTM were not detected in air samples collected in House 4 shower after dehumidification. Cartoon created using Biorender.

To reduce the overgrowth of mold observed on plates during the first round of sampling, air samples were directly impacted onto agar plates supplemented with malachite green for the second sampling event. Among the mold reduced plates, three of the 50 were NTM positive (6.0%) (Additional Fig. 4). *M. chelonae* was identified from the post shower air sample from House 2 incubated at 30 °C, but not from any other sample tested. Like the results shown in Table 1 for *M. chelonae*, we observed the disappearance of *M. intracellulare* from the shower air after dehumidification despite it being a colonizer of the showerhead (House 4) and becoming aerosolized after an 8 min shower episode (Fig. 2). For both *M. chelonae* (Table 1) and *M. intracellulare* (Fig. 2), reduction of NTM corresponded with higher RH levels after showering and before dehumidification, with no detection of NTM from shower air after dehumidifier use.

## Discussion

To our knowledge, this is the first study to evaluate the usefulness of reducing RH in showers as a possible environmental intervention for NTM such as *M. chelonae* and *M. intracellulare.* Across both sampling events, one in five homes (20%) showed NTM recovery from shower and air samples. Both *M. chelonae* and *M. intracellulare* have been previously identified from a variety of environmental sources in Hawai’i including showerhead biofilms [6, 7], but this is the first report of viable isolate recovery from air samples among households in Hawai’i. PCR studies performed using air samples similarly collected from public buildings in China demonstrated a 7.3–23.6% mycobacterial detection rate [12]. Water-to-air transfer of NTM has also been reported from a Virginia river by placing inverted petri dishes 10 cm above river water surfaces [13].

Like *M. abscessus, M. chelonae* is a pathogenic, rapid growing, and antibiotic resistant mycobacterium found in soil, dust, and water. *M. chelonae* is responsible for localized cutaneous and soft tissue disease in immunocompetent hosts [14] and lung disease in people with cystic fibrosis [15]. In Oregon, *M. chelonae* prevalence was 0.2 cases per 100,000 persons [16]. Like *M. avium, M. intracellulare* is a slow growing mycobacterium also ubiquitous in the environment and a leading cause of NTM lung disease worldwide [17, 18]. Thus, our recovery and identification of these relevant NTM species is significant and aligns with prior observations.

The reasons why House 1 showed positive NTM cultures in the first sampling event and negative cultures in the second event and why House 4 showed positive NTM cultures in the second event and negative cultures in the first event are unknown. This discrepancy may be attributed to each sampling event representing a single snapshot in time, highlighting the transient nature of showerhead NTM biofilms across time.

Overall results from two sampling periods suggest reducing RH in showers lowers NTM transfer from showerhead biofilms to the air after a showering episode. Other suggestions to reduce NTM exposures are anecdotal and include avoiding long showers or using showerheads with small pore sizes and avoiding dust inhalation [19]. Studies show raising hot water heater temperatures, disinfecting showerheads, and using UV filtration systems or point of use filters also reduce NTM [20, 21]. Conversely, ultrasonic humidifiers generate high density aerosols that are rich in NTM [8] and should be avoided. In lieu of using municipal water systems, well water is suggested as it contains lower NTM counts [19]. We recommend large-scale, prospective case-control community trials should be performed to determine the most useful environmental intervention strategies.

This study has limitations. First, our small sample size and the limitation that samples were collected from homes in southeast O’ahu, and not from homes on neighboring islands or elsewhere preclude making generalizable conclusions on the role of RH in NTM aerosolization in showers. Second, mold was common among our air samples which may have interfered with efficient NTM recovery and limited NTM identification. Since SAS units impact air directly onto plates, we were unable to further disinfect air samples. Our strategy of using culture plates with malachite green reduced mold overgrowth, but other methods for reducing fungal contamination should be investigated.

In conclusion, this study suggests running a shower is a possible source of NTM aerosolization and using a commercially available household dehumidifier during showering in conjunction with opening bathroom doors and windows may be an effective intervention to reduce environmental NTM exposures.

### Electronic supplementary material

Below is the link to the electronic supplementary material.

Supplementary Material 1

## Data Availability

The data that support the findings of this study are openly available in NCBI GenBank accession numbers OR438667-OR438674 (Round 1) and accession numbers OR428696-OR428698 (Round 2).

## Declarations

### Author information

MXK is a senior at ‘Iolani School in Honolulu, Hawai’i. With a cumulative weighted GPA of 4.5, MXK is a five-time recipient of ‘Iolani’s Top Scholar Award. MXK recently worked at the National Institutes of Health (NIH) as a Summer Cancer Research Training Fellow and plans to pursue a career in translational research and hopes to eventually obtain an MD & PhD. Outside of the lab and classroom, MXK is a leader within the community and serves as an ‘Iolani School Proconsul.
