# Supplementary material for: Shower dehumidification to reduce nontuberculous mycobacteria aerosolization
Source: BMC Res Notes. 2024 Mar 28;17:91. doi: 10.1186/s13104-024-06751-6 (PMC10976743; doi:10.1186/s13104-024-06751-6)
Supplement: Supplementary file 1 — Supplementary Material 1 [file 13104_2024_6751_MOESM1_ESM.pdf]

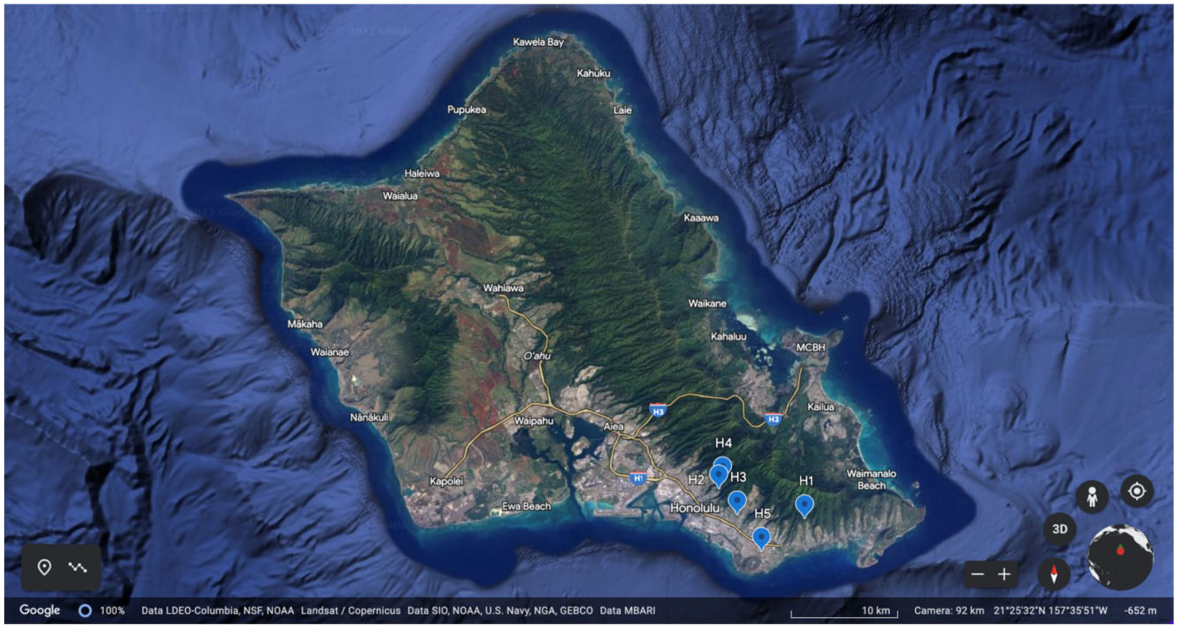

Additional Figure 1. Location of O'ahu Sampling Sites. A total of five showers in five homes were included in this study; all located on the island of O'ahu, Hawai'i in known areas of the island with high moisture and humidity including homes on the windward side of the island and Manoa Valley.

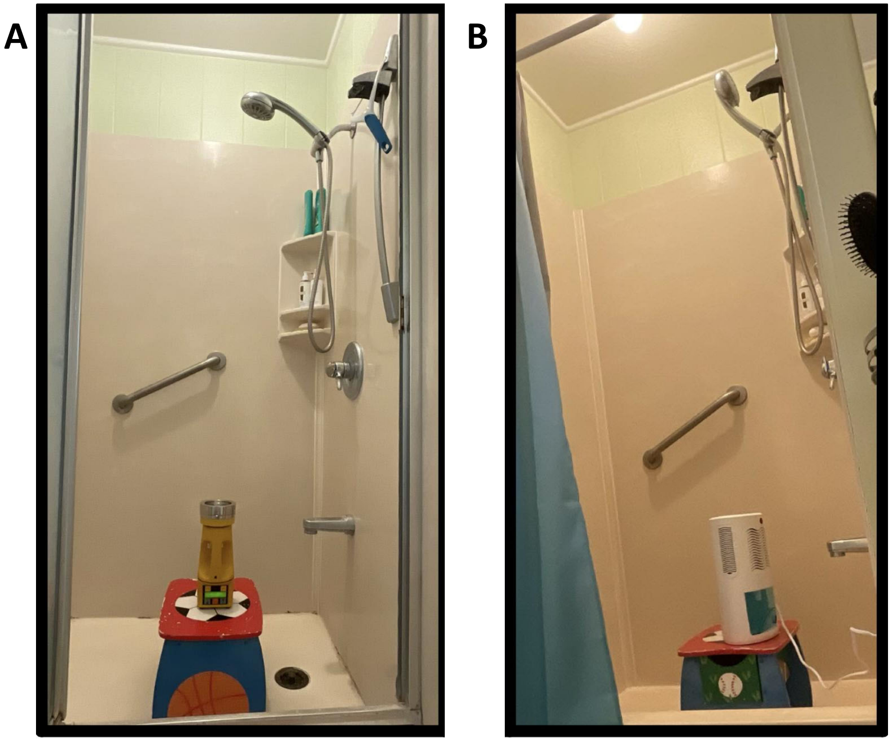

Additional Figure 2. Example images showing equipment set up in a shower. Both the SAS Sampler (left) and household dehumidifier (right) were placed 6.5’ directly below the showerhead. Special attention was paid to ensure the SAS and dehumidifier were placed in the same location.

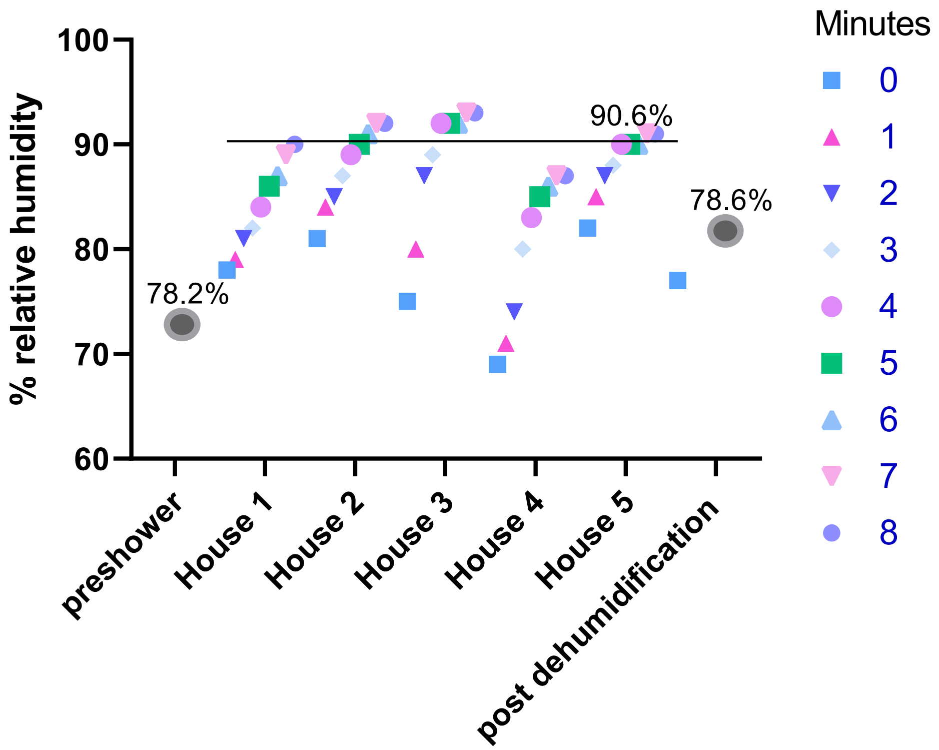

Additional Figure 3. RH levels increase over an 8min recording period after shower runs, but lowers after dehumidification, second sampling event. As in Figure 1, ambient RH levels was measured using a hygrometer before the shower was turned on and averaged across the five homes sampled ("preshower"). Shower RH levels were recorded at each of the five homes at 1min intervals for 8min while the shower was running. A final RH measurement was recorded for each shower after dehumidification ("post dehumidification"), the average value is shown.

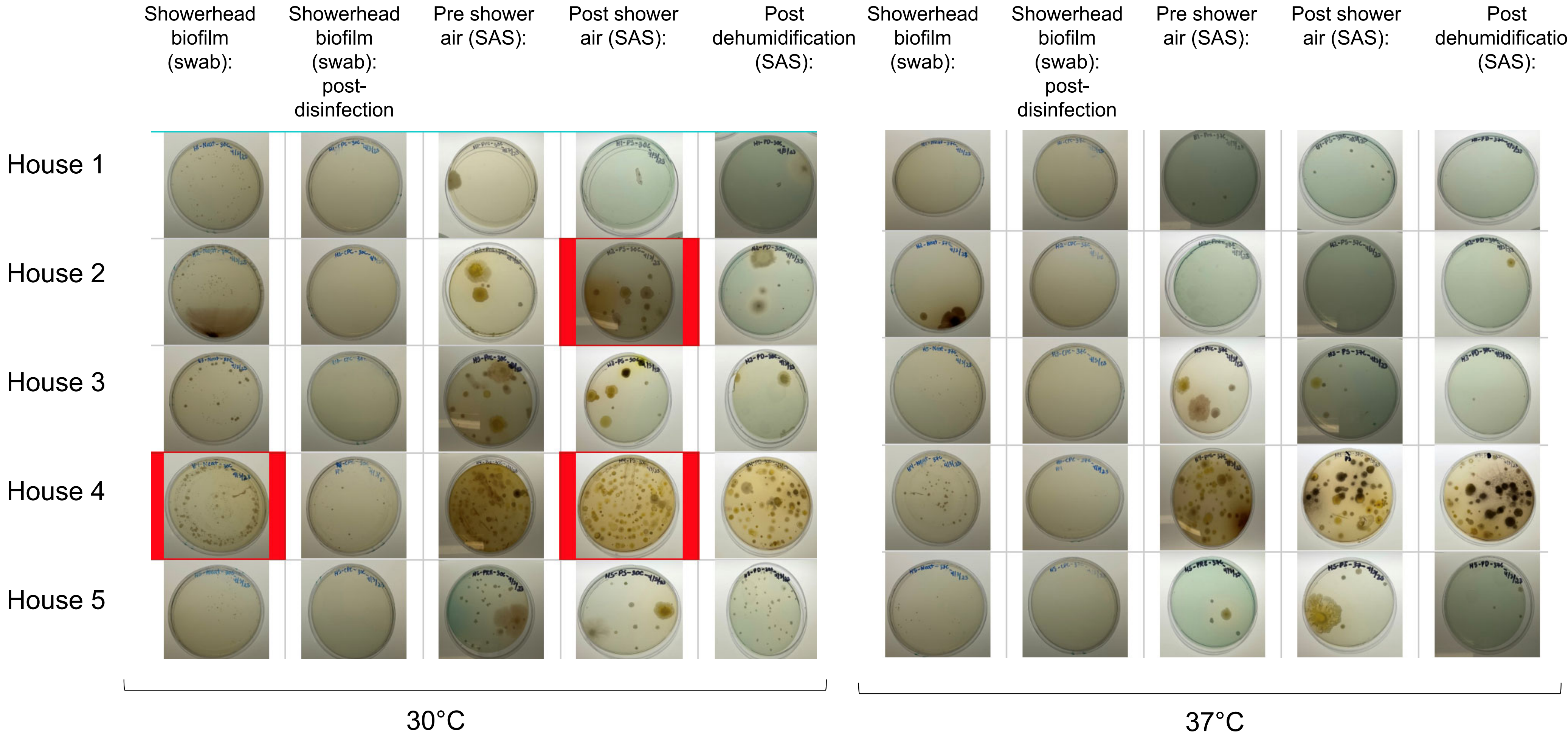

Additional Figure 4. Collective plate photos from sampling event 2. Showerhead biofilm samples were plated on Middlebrook 7H10+OADC plates before and after CPC disinfection. Air samples were impacted onto Middlebrook 7H10+OADC plates supplemented with 1% malachite green. All plates were incubated at 30°C or 37°C for 17 days. NTM-like colonies were picked from a variety of plates, but only 3 plates (red box) were identified as NTM positive by 16s rRNA and *rpoB* sequencing.
